# Differential Expression of Steroid Hormone Receptors and Ten Eleven Translocation Proteins in Endometrial Cancer Cells

**DOI:** 10.3389/fonc.2022.763464

**Published:** 2022-03-11

**Authors:** Vishakha Mahajan, Palak Gujral, Lekha Jain, Anna P. Ponnampalam

**Affiliations:** ^1^The Liggins Institute, University of Auckland, Auckland, New Zealand; ^2^Department of Physiology, Faculty of Medical and Health Sciences, University of Auckland, Auckland, New Zealand; ^3^Department of Pharmacology, Faculty of Medical and Health Sciences, University of Auckland, Auckland, New Zealand; ^4^Department of Obstetrics and Gynaecology, Faculty of Medical and Health Sciences, University of Auckland, Auckland, New Zealand

**Keywords:** gene expression, steroid hormones and receptors, endometrial cancer cells, ten eleven translocation (TET proteins), DNA hydroxymethylation (5hmC)

## Abstract

Steroid hormones govern the complex, cyclic changes of the endometrium, predominantly through their receptors. An interplay between steroid hormones and epigenetic mechanisms controls the dynamic endometrial gene regulation. Abnormalities in expression of genes and enzymes associated with steroid hormone signaling, contribute to a disturbed hormonal equilibrium. Limited evidence suggests the involvement of TET (Ten Eleven Translocation)-mediated DNA hydroxymethylation in endometrial cancer, with some data on the use of TET1 as a potential prognostic and diagnostic biomarker, however the mechanisms guiding it and its regulation remains unexplored. This study aims to explore the changes in the expressions of TETs and steroid hormone receptors in response to estrogen and progesterone in endometrial cancer cells. Gene expression was examined using real-time PCR and protein expression was quantified using fluorescent western blotting in endometrial cancer cell lines (AN3 and RL95-2). Results indicate that TET1 and TET3 gene and protein expression was cell-specific in cancer cell-lines. Protein expression of TET1 was downregulated in AN3 cells, while TET1 and TET3 expressions were both upregulated in RL95-2 cells in response to estrogen-progesterone. Further, a decreased AR expression in AN3 cells and an increased ERα and ERβ protein expressions in RL95-2 cells was seen in response to estrogen-progesterone. PR gene and protein expression was absent from both cancer cell-lines. Overall, results imply that expressions of steroid hormones, steroid-hormone receptors and TETs are co-regulated in endometrial cancer-cells. Further studies are needed to interpret how these mechanisms fit in with DNMTs and DNA methylation in regulating endometrial biology. Understanding the role of TETs and hydroxymethylation in steroid hormone receptor regulation is crucial to comprehend how these mechanisms work together in a broader context of epigenetics in the endometrium and its pathologies.

## Background

A two-way communication between epigenetic mechanisms and steroid hormones is crucial for the healthy functioning of the endometrium. Estrogen and progesterone, secreted by the ovaries, execute their functions predominantly *via* steroid hormone receptors - estrogen receptor (ER) and progesterone receptor (PR). Transcriptional regulation of steroid hormone receptors in the endometrium is partly controlled by epigenetic factors like DNA methylation and hydroxymethylation (1–5). DNA methylation yields 5-methylcytosine (5mC), making for one of the most important forms of epigenetic modification in the mammalian DNA (6). However, the modification of DNA from 5C (5-Cytosine) to 5mC can be actively or passively reversed *via* the process of DNA de-methylation. The DNA de-methylation cascade consists of the initial oxidation of 5mC into 5-hydroxymethylcytosine (5hmC) followed by a series of additional oxidation steps (7, 8). 5hmC is identified as an independent epigenetic modification that can alter gene expression and might be important in epigenetic reprogramming (8). The active de-methylation process is catalysed by ten-eleven translocation (TET) enzymes, making them an essential component in epigenetic machinery. Dysregulation of TETs and subsequent 5hmC marks have been implicated in endometrial diseases such as endometrial cancer and endometriosis. (9, 10). Knockout study models have previously been used to establish the function of TETs in various tissues and cells including the maintenance of reproductive axis and epigenetic reprogramming (11–15). DNA methylation is known to be involved in maintaining successful steroid hormone signaling by regulating steroid hormone receptors (16). On the other hand, estrogen and progesterone can influence mRNA and protein expression of DNA Methyltransferases (DNMTs), thereby affecting methylation patterns (17–19).

In the normal endometrium, increasing estrogen levels during the proliferative phase, lead to an increase in the expression of estrogen (ER), progesterone (PR) and androgen receptors (AR) (20). This is followed by an antagonistic progesterone action which is mediated by the increased levels of progesterone receptors (21, 22). The interplay between estrogen and progesterone implies that while estrogen action aids in upregulating steroid receptors in the endometrium, progesterone action downregulates them (20). Since the maintenance of this steroid hormone equilibrium is essential to endometrial biology, abnormal regulation of steroid hormone receptor expression can contribute to endometrial pathologies (23–28). Previously, it has been suggested that TETs and DNMTs could potentially be inversely regulated by steroid hormones, with epithelial cells being more sensitive and responsive to steroid hormone treatments (29). This study is aimed at mimicking the hormonal influences seen during the menstrual cycle *in vitro*, to explore the mechanisms involved in the regulation of TETs and steroid hormone receptors in endometrial cancer cells. Four steroid hormone receptors– Estrogen receptors alpha (ERα) and beta (ERβ), Progesterone receptor (PR) and Androgen Receptor (AR) along with TETs were examined to assess the role of steroid hormones in their transcriptional and translational regulation.

## Material and Methods

### Preparation and Treatment of Cell Lines

Endometrial adenocarcinoma cell lines, AN3 (ATCC^®^ HTB-111^™^) and RL95-2 (ATCC^®^ CRL-1671^™^) were used for this study. All cells were cultured either in phenol-free DMEM or RPMI medium, supplemented with 10% charcoal stripped FBS (CS-FBS) as well as 1% of penicillin-streptomycin antibiotic (ThermoFisher Scientific, USA). The cells were then cultured in a humidified atmosphere with 5% CO_2_ at 37°C until confluent. Cells were then plated in twelve-well culture plates and upon 80% confluence, they were primed with 0.01μM of β-estradiol (E treatment) for 24h. Followed by the addition of progesterone (1μM) to the estrogen primed (EP treatment) wells for 24, 48 and 72h. Ethanol at a concentration of less than 0.01% was used as control (C treatment). The treatment solutions were prepared using commercially available powdered concentrates (Sigma- Aldrich, USA) and dissolved in analytical grade ethanol. The final concentrations were prepared in culture media and stored at -80°C until further use.

### RNA Extraction

Trizol^®^ reagent (Life Technologies, NZ) was used to extract Total RNA. 1ml of Trizol^®^ was added per well and cells were detached using a cell scrapper. The cells were homogenized and treated according to the manufacturer’s instructions. Using the protocol provided, chloroform (0.2ml/1ml of Trizol^®^) was added to the samples and vigorously shaken and incubated for 3minutes at room temperature. After a 15minute centrifugation (12000xg) at 4°C, isopropanol (0.5ml/1ml of Trizol^®^) was added to the aqueous phase and incubated for 20minutes on ice. Followed by another centrifugation under similar conditions, the RNA pellet was obtained and washed in 70% ethanol with additional 10minute centrifugations, twice. The pellet was air dried at room temperature and suspended in DEPC treated water. The concentration and quality of RNA was assessed using the NanoPhotometer^®^ (Implen, Germany). An OD260/280 ratio of 1.8 to 2.0 was considered quality RNA.

### Reverse Transcription and Quantitative RT-PCR

As directed by the manufacturer’s instruction manual, 1µg of RNA was treated with 1µl of 10xDNase and DNAse Buffer each and made up to 10µl with DEPC-water. After a 15minute incubation at room temperature, 1µl of EDTA was added to each reaction tube and incubated at 65°C for 10minutes. Reverse transcription into single-stranded cDNA was performed using High Capacity cDNA Reverse Transcription Kit (Applied Biosystems, USA). According to the manufacturer’s instructions, each tube was mixed with Reverse Transcriptase Buffer, Random primers, Deoxynucleotide Mix and Reverse Transcriptase and made it up to a total reaction volume of 20μl. Using the BioRad DNA Engine^®^ Peltier Thermal Cycler under the following conditions: 10minutes at 25°C, 120minutes at 37°C and 5minutes at 85°C, reverse transcription was performed. The resulting cDNA was diluted with nuclease-free water (1:10) and used for real-time PCR. PCR analysis was performed and conducted using QuantStudio (Applied Biosystems, USA) as previously described (29). Primers for *TET1, TET2, TET3, RPL13a, YWHAZ* and *RPLO* (**Table 1**) were obtained from Primer Bank (30–33). Primers for *ERα*, *ERβ* and *PGR* were the PrimeTime predesigned qPCR Assays (IDT) (**Table 1**). Primer (**Table 1**) for *AR* was obtained from a previously published study by Kamal et al. (34). Gene expression analysis was done using the comparative CT method (ΔΔCT method) (35). All the results were normalized to the geomeans of the three reference genes- *YWHAZ, RPL13a* and *RPLO* as described previously (29).

**Table 1:** Primer Sequences used for qRT-PCR.

Gene	Sense	Antisense
*TET1*	CAGAACCTAAACCACCCGTG	TGCTTCGTAGCGCCATTGTAA
*TET2*	GAGCAGGTCCTAATGTGGCAG	GCTCGCTCCCGCACCAA
*TET3*	TCCAGCAACTCCTAGAACTGAG	AGGCCGCTTGAATACTGACTG
*ERα*	CCCACTCAACAGCGTGTCTC	CGTCGATTATCTGAATTTGGCCT
*ERβ*	AGCACGGCTCCATATACATACC	TGGACCACTAAAGGAGAAAGGT
*PGR*	ACCCGCCCTATCTCAACTACC	AGGACACCATAATGACAGCCT
*AR*	AGGATGCTCTACTTCGCCCC	CTGGCTGTACATCCGGGAC
*RPL13a*	GCCCTACGACAAGAAAAAGCG	TACTTCCAGCCAACCTCGTGA
*YWHAZ*	CCGTTACTTGGCTGAGGTTG	CAGGCTTTCTCTGGGGAGTT
*RPLO*	AGAAACTGCTGCCTC ATATCCG	CCCCTGGAGATTTTA GTGGTGA

### Protein Extraction

AN3 and RL95-2 cells were extracted from culture plates using RIPA lysis and extraction buffer (ThermoFisher Scientific, USA). According to the instructions provided, culture media was aspirated and 1ml/well of cold RIPA buffer was added to lyse the cells. Halt™ Protease Inhibitor Cocktail (EDTA-Free (100X)) (Thermo Fisher Scientific) was also added (20µL per 1mL of RIPA lysis buffer). The plate was then incubated on ice for 5minutes with intermittent swirling for uniform spreading of the buffer. The lysate was gathered using a cell scraper and transferred to a 2ml tube. The samples were then centrifuged at 14000xg for 15 minutes and the supernatant protein was collected and stored at -80°C for further analysis.

### Fluorescent Western Blot

Western blot analysis was performed to evaluate the expression of TETs and steroid hormone receptor proteins post-treatment. Protein extracted from the cells were loaded on a 3-8% NuPage^™^ Tris Acetate gel (Invitrogen, USA) and transferred onto a 0.45 micron pored fluorescent polyvinyl difluoride membrane (Fl-PVDF), (Millipore, USA). The protocol for a wet transfer was followed according to the manufacturer’s instructions using 20X NuPage^™^ Transfer Buffer (Invitrogen, USA). Once the proteins were transferred onto the membrane, it was stained and washed with Revert^™^ total protein stain and wash solution respectively, (Licor Biosciences, USA). The membrane was imaged at 700nm Odyssey^®^ imaging system and blocked using Intercept Blocking Buffer (Licor Biosciences, USA) for an hour at room temperature. Primary antibodies were diluted according to **Table 2** in the blocking buffer. Following which, the membrane was incubated in primary antibody and left overnight at 4°C and conjugated with secondary antibodies the next day. The membrane was washed thrice using 1XTBST (Tris-buffered saline with Tween-20) with 5minute intervals and incubated with the secondary antibody for 1hr at room temperature. The membrane was washed, dried, and imaged for 10minutes at 800nm channel using Odyssey^®^ imaging system (Licor Biosciences, USA). All the primary antibodies (**Table 2**) used for this experiment were from Thermo Fisher Scientific and the secondary antibodies were from Licor Biosciences - donkey anti-mouse (P/N: 926-32212) or goat anti-rabbit (Catalog# P/N: 926-32211) IRDye^®^ 800CW depending on primary antibody reactivity. Protein expression for all samples were normalized to the total protein stain for each blot. Target protein bands were normalized against the total protein transferred per lane. Total protein signal (TPS) was used to calculate the proteins in each lane and the normalization factor. The formulas used for each calculation are below:


$$\text{Lane Normalization Factor}=\frac{TPS\;for\;each\;lane}{TPS\;from\;the\;lane\;with\;the\;highest\;TPS}$$

$$\text{Normalization Signal}=\frac{Target\;band\;signal}{Lane\;normalization\;factor}$$

**Table 2:** Details of primary antibodies used and their dilutions.

Primary Antibody	Host	Dilution	Catalogue Number
ERα	Mouse	1:500	MA514501
ERβ	Mouse	1:1000	PA1311
PR	Mouse	1:500	MA1410
AR	Mouse	1:200	MA513426
TET 1	Mouse	1:400	MA5-16312
TET 2	Rabbit	1:300	PA5-76801
TET 3	Rabbit	1:200	PA5-31860

The normalized signal for each sample was calculated to be used for relative quantitative comparison. The x-axis demonstrated the fold change that was normalized to the control and was plotted against the treatment stage (y-axis) for each sample.

### Statistical Analysis

GraphPad Prism 8 (GraphPad Software, La Jolla, CA) and IBM SPSS version 27.0 (Armonk, NY) were used to analyze the data obtained. Statistical tests included one-way analysis of variance (ANOVA) and paired t-test to determine significance (P<0.05 was considered statistically significant; P ≤ 0.1 was considered as approaching significance). All the Graphs were generated using GraphPad Prism 8 (GraphPad Software, CA).

## Results

### Gene Expression of TETs and Steroid Hormone Receptors in AN3 Cell Lines in Response to Steroid Hormone Treatment

*TET1* was significantly downregulated (p=0.0479) post the initial 24 hour estrogen treatment, followed by a significant increase in response to a combined estrogen-progesterone treatment for 24 hours (p=0.0361). Prolonged exposure to combined estrogen-progesterone for 48 and 72 hours resulted in a significant reduction of *TET1* gene expression (p=0.0302). *TET2* and *TET3* did not display any significant in response to any treatments, although there was a significant increase in *TET2* between combined estrogen-progesterone treatment from 24 to 48 hours (p=0.0276). No significant changes in steroid hormone receptor expression were observed in AN3 cells in response to any treatments.

### Protein Expression of TETs and Steroid Hormone Receptors in AN3 Cell Lines in Response to Steroid Hormone Treatment

TET1, 2 and 3 proteins in AN3 cells exhibited no changes during estrogen only treatment as observed in **Figure 2**. However, differential expression was observed when treated with combined estrogen-progesterone for 24, 48 and 72 hours. TET1 protein expression displayed a decreasing trend when exposed to 72 hours of combined estrogen-progesterone treatment (p=0.1). Conversely, TET3 protein expression displayed a trend toward increasing upon 72 hours of estrogen-progesterone treatment (p=0.1). Furthermore, there was a trend towards an increase in TET2 protein expression in response to 24-hour estrogen-progesterone treatment, approaching significance (p=0.1). Protein expression for steroid hormone receptors revealed no significant changes in ERβ expression. However, AR protein expression was consistently downregulated during treatment with estrogen-progesterone for 24 (p=0.08), 48 (p=0.059) and 72 (p=0.09) hours (**Figure 3**). No bands for ERα, PRA and PRB proteins were not detectable by western blotting in AN3 cells under any treatments.

### Gene Expression of TETs and Steroid Hormone Receptors in RL95-2 Cell Lines Upon Steroid Hormone Treatment

Gene expressions for all three *TETs* varied significantly across different treatments (p< 0.0001) in RL95-2 cells. However, no statistical significance was found between individual treatment groups in comparison to the control. Gene expression for *ERα, PR* or *AR* were not detected in RL95-2 cells. However, *ERβ* gene expression was prominent in hormone treated RL95-2 cells with significant changes between control and treatments (p<0.0001), as revealed by one way ANOVA with no significant differences between individual treatment groups (**Figure 4**).

### Protein Expression of TETs and Steroid Hormone Receptors in RL95-2 Cell Lines Upon Steroid Hormone Treatment

Protein expression of TETs varied across different treatments as shown in **Figure 5**. TET1 (p=0.01) expression was significantly decreased and a similar trend towards a decrease in TET3 (p=0.1) expression was also seen in response to estrogen only treatment. There was a significant increase in TET3 (p=0.019) and a trend towards an increase in TET1 (p=0.1), when treated with estrogen-progesterone for 72 hours. In response to 48 hours of estrogen-progesterone treatment, a significant increase in TET3 (p=0.02) and a trend towards reduction in TET1 (p=0.1) expression was observed. TET2 protein expression was significantly upregulated during estrogen only treatment (*p=0.059). The protein expression of ERs revealed a differential and treatment dependent regulation as shown in **Figure 6**. There was a trend towards an increase in ERα protein (p=0.1) expression in response to 24 hours of estrogen-progesterone treatment, and ERβ expression (p=0.1) in response to estrogen only treatment which stayed consistent across treatments with significant increase seen in response to 72 hours of a combined estrogen-progesterone treatment (p=0.038). Very faint bands for AR were observed with no significant differences between treatments. PRA and PRB were not detected in RL95-2 cells even in response to treatment.

## Discussion

DNA methylation and hydroxymethylation are crucial components of the epigenetic machinery. The aim of this study was to evaluate the contribution of steroid hormones in the transcriptional and translational regulation of TETs and steroid hormone receptors in endometrial cancer cells. The results indicate that the gene and protein expressions of TETs and steroid hormone receptors and their response to steroid hormones is cell-specific and differ between AN3 and RL95 cells.

### TET and Steroid Hormone Receptor Regulation in AN3 Cell Line

**Figure 1:**
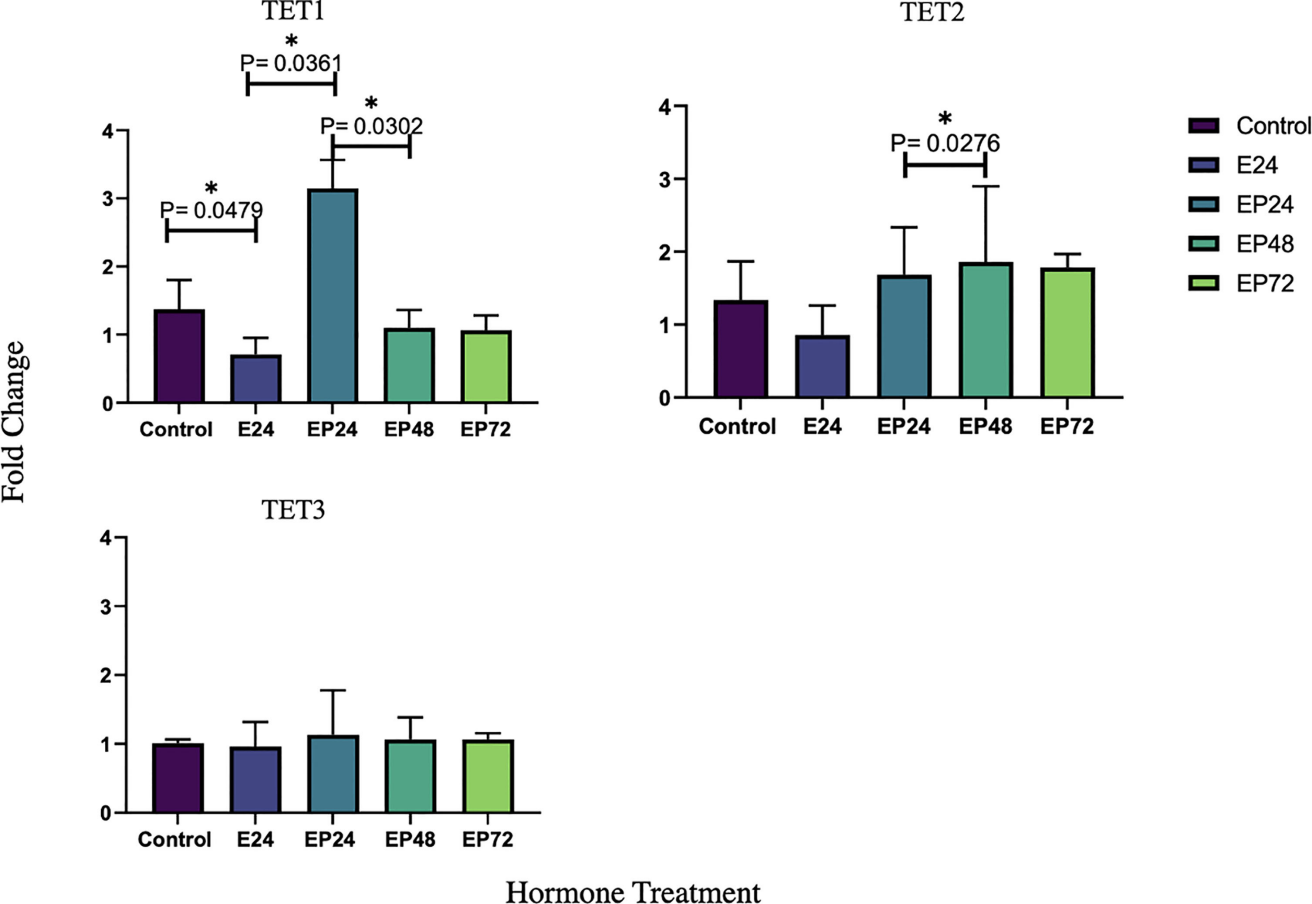
Relative TET1 TET2 and TET3 mRNA expression in response to steroid hormone treatment in AN3 cells. The y-axis shows the fold change of mRNA levels following different treatments compared with control, all results corrected against geo-mean expressions of three reference genes - YWHAZ, RPLO and RPL13a. The x-axis shows different treatment groups. E24 = 24h Estrogen; EP24, EP48 and EP72 = both Estrogen + Progesterone for 24, 48 and 72h. Data are presented as mean ± SEM *p < 0.05. P ≤ 0.1 was considered as approaching significance. The experimental setup included three independent sets of cell culture experiments (n = 3) and triplicates of each sample for the RT-PCR.

Endometrial pathologies such as endometrial cancer are steroid dependent disorders. Steroid hormones guide the fluctuating epigenetic patterns, allowing genes to be expressed or repressed during the menstrual cycle (16). In AN3 cells, *TET1* transcription was significantly downregulated when exposed to estrogen for 24 hours. This was then marked by a significant increase when exposed to a combined estrogen-progesterone treatment for 24 hours. However, a further prolonged estrogen-progesterone treatment for 48 and 72 hours resulted in a significant decrease in *TET1* mRNA expression (**Figure 1**). *TET1* gene expression in AN3 cells, was responsive to the slightest change in treatment, which could indicate its sensitivity to subtle hormonal changes.

TET1 protein expression parallels the gene expression and is significantly downregulated when treated with estrogen-progesterone for 72 hours. According to the results of our previous study, *TET1* mRNA was upregulated during the mid-secretory phase in healthy endometrial tissues and in response to progesterone treatment in epithelial cells, *in vitro* (29). The decreased protein expression during 72 hours of estrogen-progesterone treatment, suggests a potential aberrant regulation of TET1 in AN3 cells. Data by other studies report similar findings with decreased TET1 mRNA and protein expression in endometrial cancer tissues compared to normal (9, 36). It has been suggested that overexpression of DNMT3a and DNMT3b contribute to hypermethylation of *ERα* and *PR*, subsequently silencing these genes in endometrial cancer (37). TETs mediate epigenetic alterations *via* DNA de-methylation, a process where they actively remove the methyl group, to activate gene expression (38). Downregulation of TET1 gene and protein expression, could be associated with the abnormal inactivation of ERα and PR seen in endometrial cancer tissues (37, 39, 40). While other studies have reported mRNA expression of *ERa* in AN3 cells at the basal level (41), neither gene nor protein expression of *ERa* or *PR* in AN3 cells in the present study. The discrepancy in the *ERa* gene expression in both the studies could be attributed to the differences in the treatment protocol used. The data obtained from this study indicates that the downregulation of TET1 in response to estrogen and progesterone could potentially be contributing to epigenetic deregulation and warrants the need for more studies to investigate its role in endometrial cancer.

**Figure 2:**
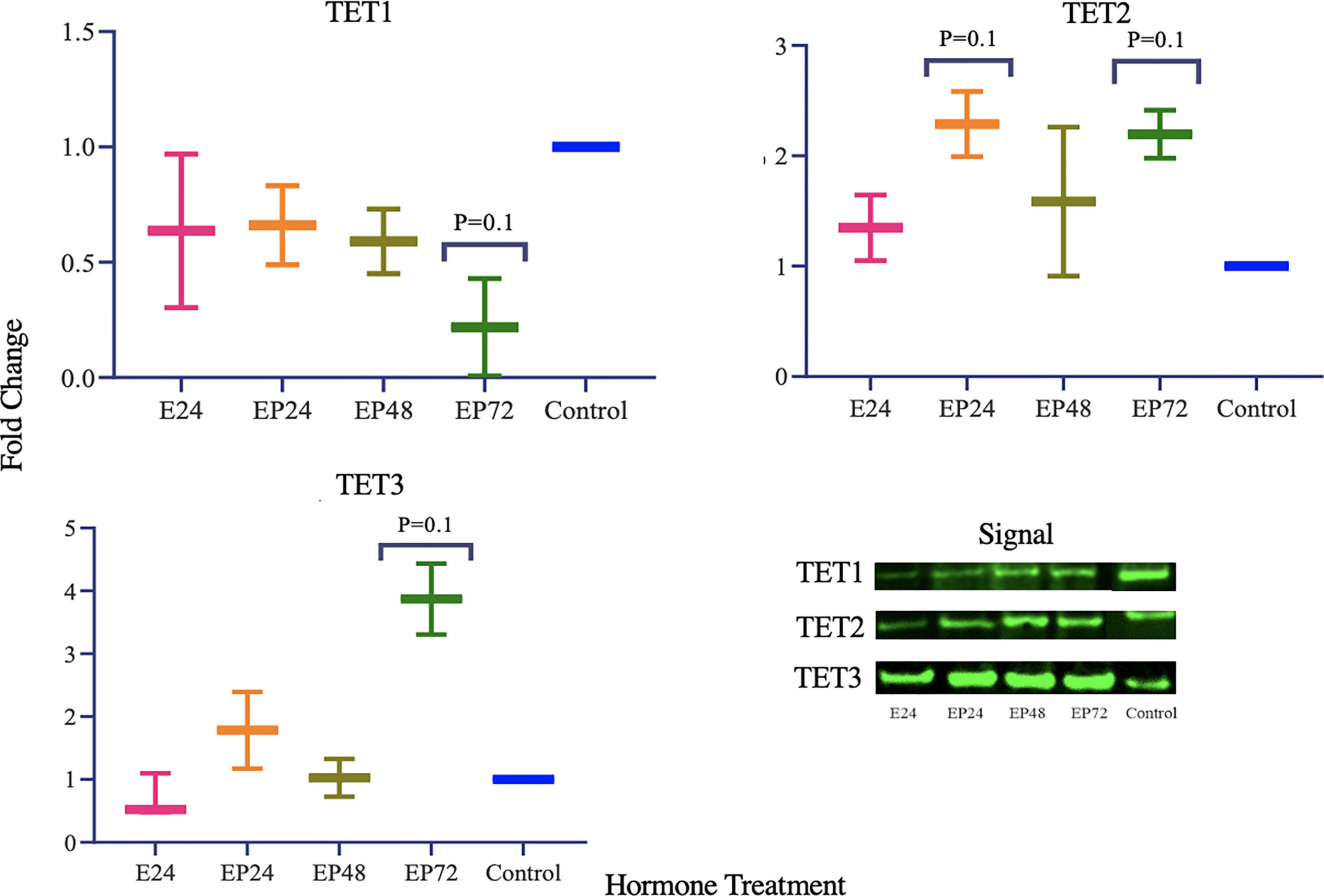
TET protein expression in response to different steroid hormone treatments in AN3 cells. A representative blot image for the particular weight band is shown next to the graph. The y-axis shows the fold change of protein levels following different treatments compared to control and x-axis shows the different treatment groups. E24, 24h Estrogen; EP24, EP48 and EP72 = both Estrogen + Progesterone for 24, 48 and 72h. Data are presented as mean ± SEM, *p < 0.05 (P ≤ 0.1 is considered as approaching significance). The experimental setup included three independent sets of cell culture experiments (n = 3) with three technical replicates for each sample.

TET2 has been previously implicated in various types of malignancies (11, 42–46). Data from the present study imply that TET2 protein expression is upregulated when exposed to a combined estrogen-progesterone treatment for 24 hours (**Figure 2**). Further, it is also seen that mRNA expression of *TET2* remains upregulated upon continued exposure to estrogen-progesterone treatment for 48 hours (**Figure 1**) in AN3 cells. *TET2* expression has been shown to be significantly reduced in severe endometrial cancer and cervical squamous cell carcinoma tissues compared to their normal counterparts (9, 47). So far to our knowledge, there are no studies that have evaluated any cell specific changes in relation to malignancy or hormonal treatment in endometrium. Our previous study reported an upregulation of *TET2* expression in non-estrogen primed, non-cancerous endometrial epithelial cells, in response to progesterone (29). Further studies are needed to fine tune the mechanisms by which TET2 might be deregulated in endometrial cancer.

While no hormonal effects on *TET3* mRNA expression were observed, TET3 protein was significantly increased during 72 hours of estrogen-progesterone treatment (**Figure 2**). This is in agreement with Cieselski et al., who also reported an increased *TET3* mRNA expression in endometrial cancer tissue biopsies (9). TET3 has been reported to be crucial in the maintenance of stem cell identity, DNA repair and overall genome stability in various tissues (48–51). Aberrations in stem cells have been implicated in the origin and progression of endometrial cancer (52, 53). Increased TET3 protein expression in cancer, could potentially indicate its involvement in abnormal stem cell regulation, contributing to progression, invasiveness and metastasis. Furthermore, reduced mRNA expression of *TET2* and *TET3* have been implicated in the induction of epithelial-mesenchymal transition in melanoma (54). This study, indicates an increased expression of TET2 and TET3 in the combined estrogen-progesterone treated samples in AN3 cells. The difference in results could be attributed to the type of cell and treatment protocols used. Collectively, it is implied that TET2 and TET3 could be involved in the differential regulation seen in cancers, however its exact association still needs to be explored further.

**Figure 3:**
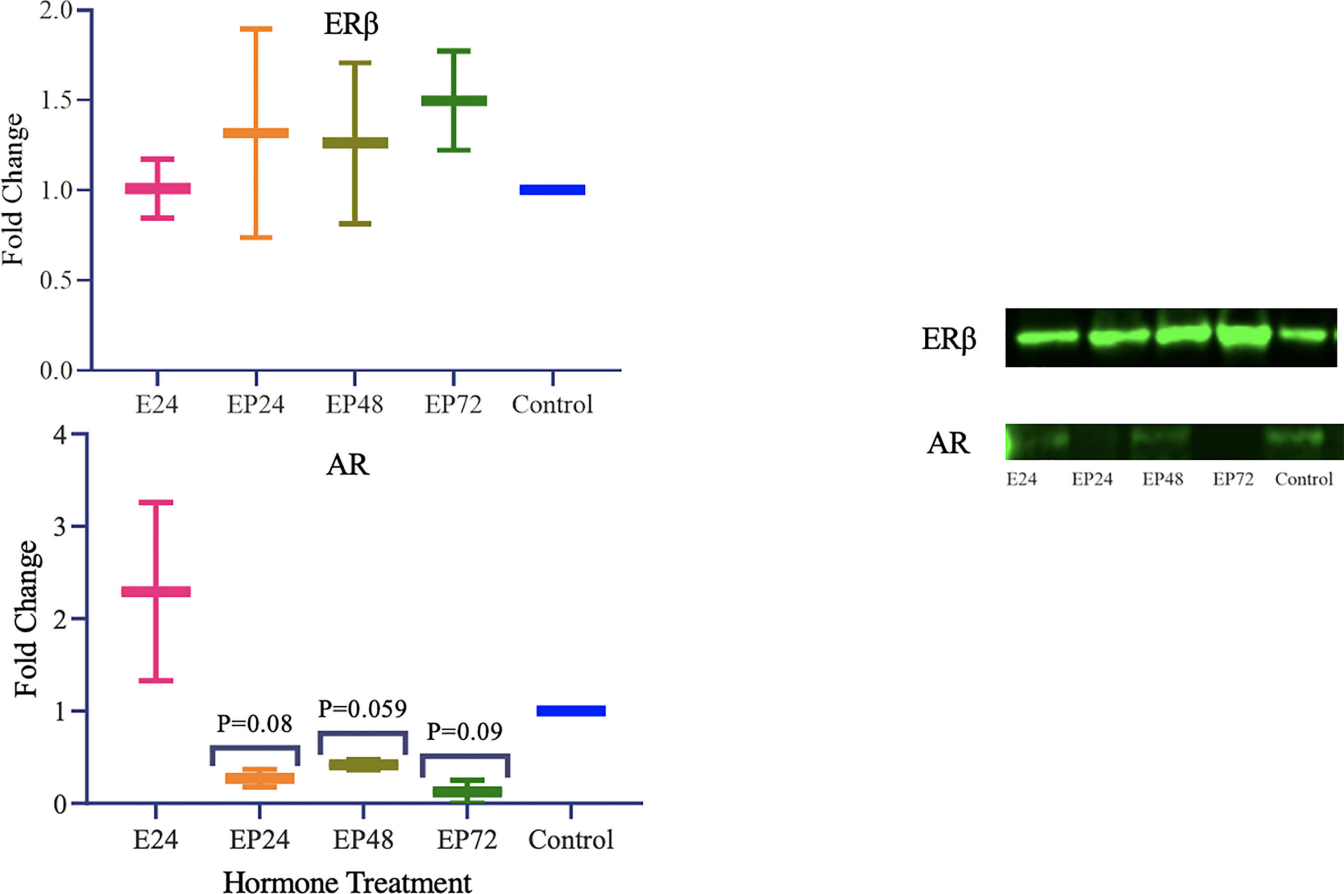
Steroid hormone protein expression in response to different steroid hormone treatments in AN3 cells. A representative blot image for the particular weight band is shown next to the graph. The y-axis shows the fold change of protein levels following different treatments compared to control and x-axis shows the different treatment groups. E24 = 24h Estrogen; EP24, EP48 and EP72 = both Estrogen + Progesterone for 24, 48 and 72h. Data are presented as mean ± SEM. P ≤ 0.1 is considered as approaching significance. The experimental setup included three independent sets of cell culture experiments (n =3) with three technical replicates for each sample.

ER and PR have been extensively studied in endometrial pathologies such as cancer and endometriosis. AR, however, is a lesser explored steroid hormone receptor in endometrial biology. Our data imply that in AN3 cells, AR protein levels were significantly downregulated when treated with estrogen-progesterone together for 24, 48 and 72 hours (**Figure 3**). AR is a known anti-estrogen, which means that it has the ability to counteract the proliferative activity of estrogen (55). In normal epithelial cells, a differential and increased expression of *AR* is reported using immunohistochemistry during the secretory phase of the endometrium (56). Downregulation of AR levels are seen in the estrogen-progesterone treated samples, could be related to the decreased TET1 mRNA and protein expression. An association between TET1 and AR has also been suggested by Dhiman et al. (57). Their study reports that TET1, AR and thymine DNA glycosylase are co-recruited to the transcription start site of the Androgen Responsive Elements (AREs) to influence gene regulation in human prostate cells (57). Thus, suggesting that TET1 could be potentially involved in the transcriptional activation of AR in endometrial biology. Moreover, it has been suggested that AR suppresses tumor growth in ER positive breast malignancies (58). The findings of this study imply that the downregulation of *AR* and absence of *PR* gene expression in AN3 cells, could be contributing to the uncontrolled proliferation, seen in endometrial cancer cells. This study suggests that steroid hormones regulate the crosstalk between TETs and steroid hormone receptors in endometrial biology. Understanding this regulation more robustly in the endometrium, could help provide novel targets for therapeutic interventions for associated pathologies.

### TET and Steroid Hormone Receptor Regulation in RL95-2 Cell Line

**Figure 4:**
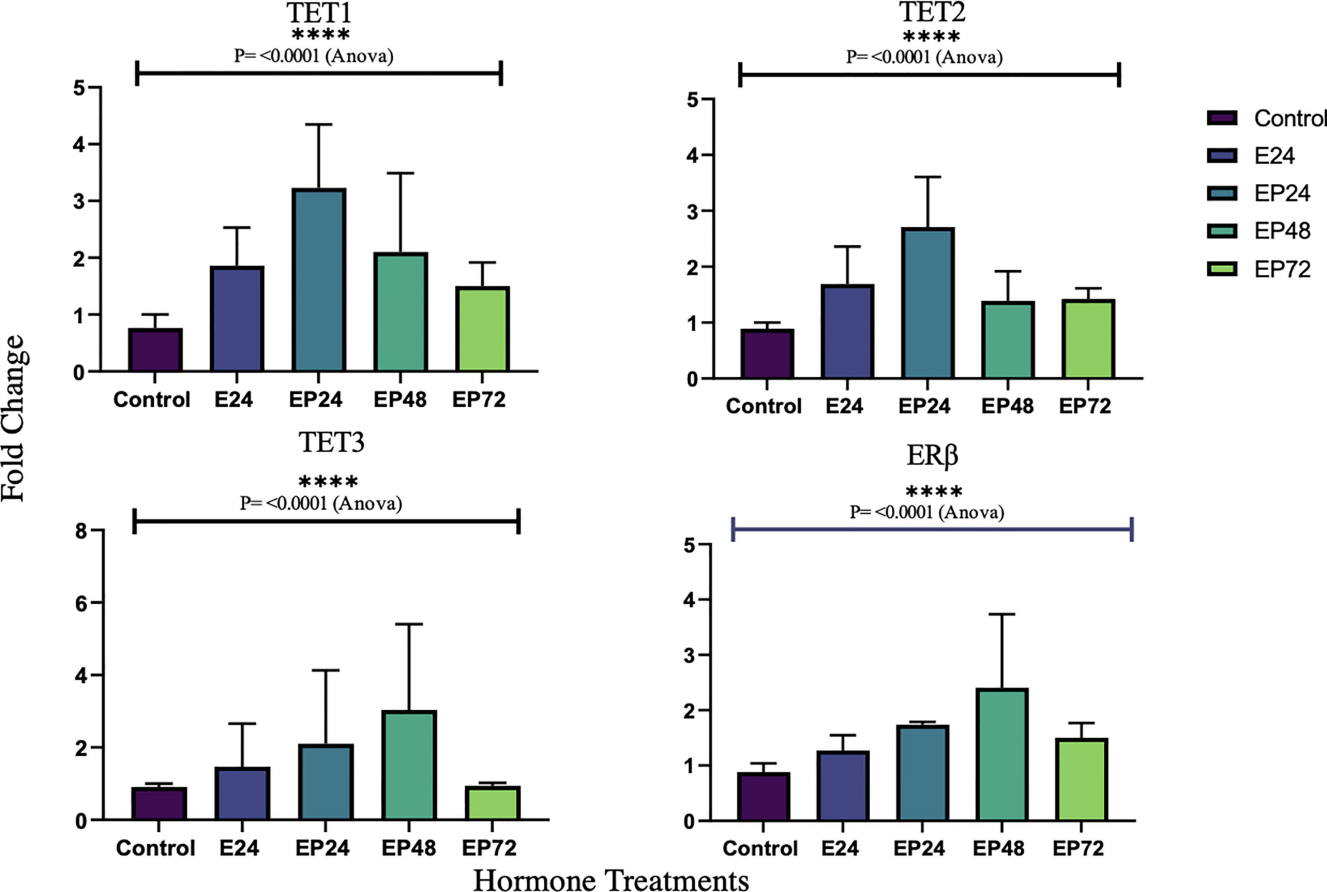
Relative TET and ERb mRNA expression in response to different steroid hormone treatments in RL95-2 cells. The y-axis shows the fold change of mRNA levels following different treatments compared with control, all results corrected against geo-mean expressions of three reference genes - YWHAZ, RPLO and RPL13a. The x-axis shows different treatment groups E24 = 24h Estrogen; EP24, EP48 and EP72 = both Estrogen + Progesterone for 24, 48 and 72h. Data are presented as mean ± SEM; ***P < 0.001; P ≤ 0.1 was considered as approaching significance. One way ANOVA test revealed significant variations in TET and ERb expression across treatments (****P<0.0001). The experimental setup included three independent sets of cell culture experiments (n =3) and triplicates of each sample for the RT-PCR.

**Figure 5:**
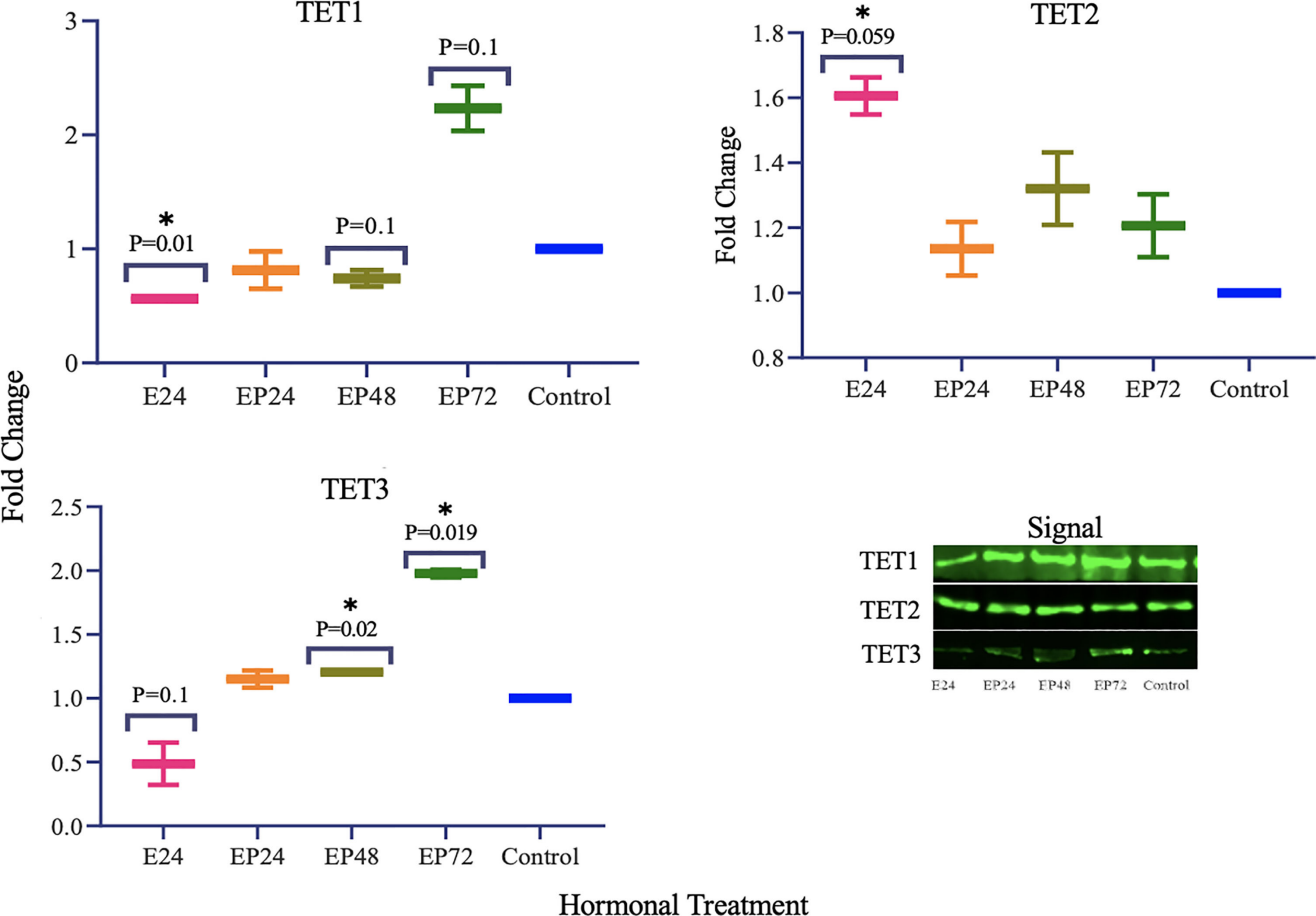
TET protein expression in response to different steroid hormone treatments in RL95-2 cells. A representative blot image for the particular weight band is shown next to the graph. The y-axis shows the fold change of protein levels following different treatments compared to control and x-axis shows the different treatment groups. E24 = 24h Estrogen; EP24, EP48 and EP72 = both Estrogen + Progesterone for 24, 48 and 72h. Data are presented as mean ± SEM, *p < 0.05, P ≤ 0.1 is considered as approaching significance. The experimental setup included three independent sets of cell culture experiments (n =3) with three technical replicates for each sample.

**Figure 6:**
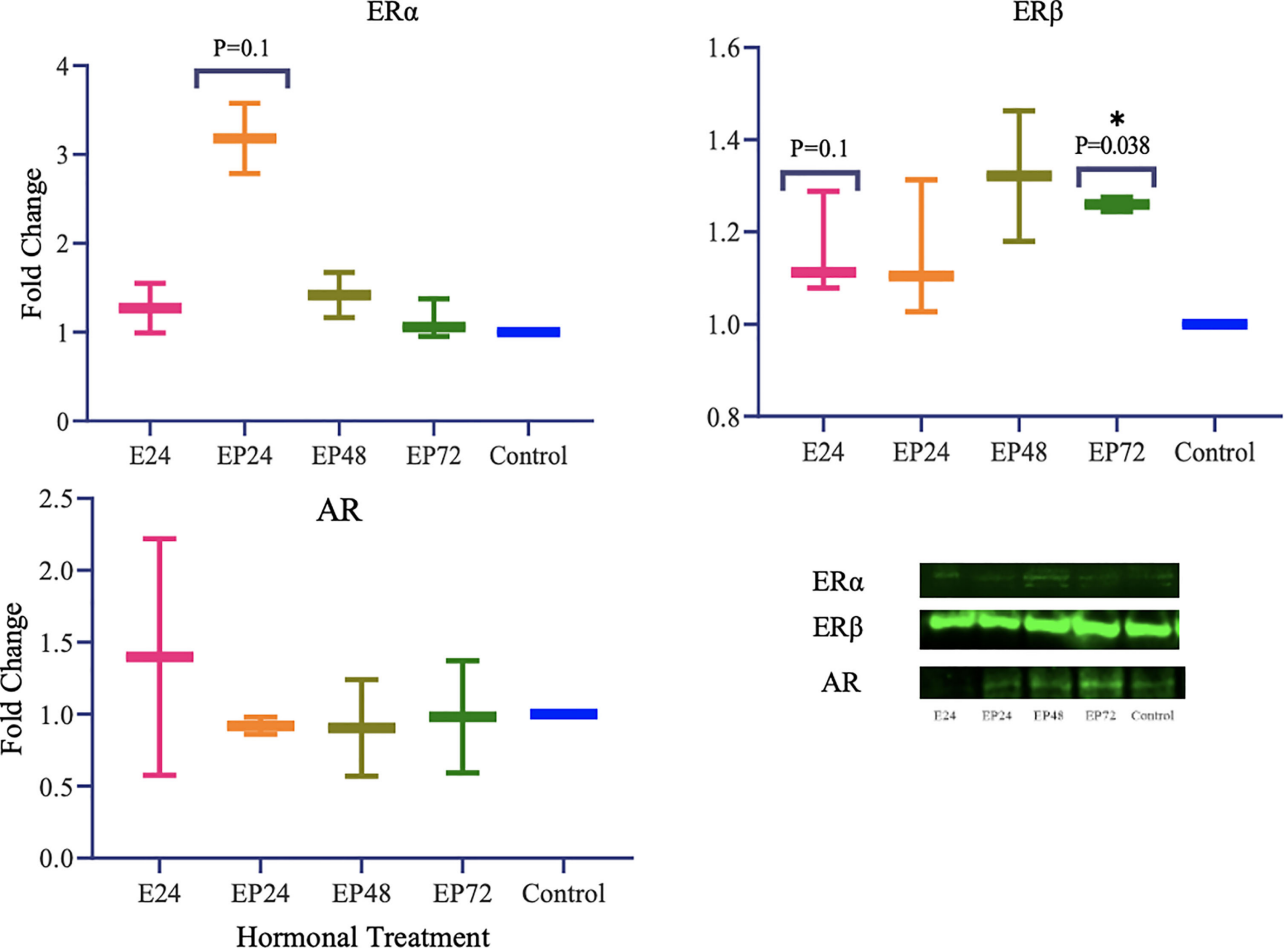
Steroid hormone protein expression in response to different steroid hormone treatments in RL95-2 cells. A representative blot image for the particular weight band is shown next to the graph. The y-axis shows the fold change of protein levels following different treatments compared to control and x-axis shows the different treatment groups. E24 = 24h Estrogen; EP24, EP48 and EP72 = both Estrogen + Progesterone for 24, 48 and 72h. Data are presented as mean ± SEM, *p < 0.05, P ≤ 0.1 is considered as approaching significance. The experimental setup included three independent sets of cell culture experiments (n = 3) with three technical replicates for each sample.

In RL95-2 cells, one way ANOVA analysis suggest a significant influence of hormones on TET mRNA expressions. However, no significant differences between individual treatments were observed. Gene expression for *TET1*, *TET2 and TET3* were highest when exposed to combined estrogen-progesterone treatments for 24 hours and 48 hours (**Figure 4**). Protein data indicated a differential expression of TETs when exposed to different treatments. TET1 and TET3 levels were significantly increased in response to 72 hour combined estrogen-progesterone treatment (**Figure 5**). mRNA and Protein upregulation of TET1 is also demonstrated in another study suggesting that the hypoxic, chronic inflammatory environment seen in endometrial cancer, can up-regulate TET1 expression and induce its downstream gene transcription (59). Upon exposure to 24 hours of estrogen, TET1 and TET3 were significantly downregulated whereas TET2 was upregulated (**Figure 5**), implying a potential difference in the regulation and function of TETs. TET2 has been reported to serve as a co-activator of ERα by de-methylating and maintaining low CpG methylation levels in breast cancer cell lines (60). This could potentially explain the significant upregulation of ERα when treated with estrogen-progesterone for 24 hours (**Figure 6**). Additionally, the upregulation of ERβ could be correlated to the increased protein expression of TET1 and TET3 in response to 72 hours of estrogen-progesterone treatment. This finding suggests a possible interplay between TETs, regulated by hormones and influencing ERα and ERβ expression in RL95-2 cells.

Conflicting studies on the mRNA expression of *ERα* and *ERβ* have been reported in endometrial cancer. While some suggest lower *ERα* expression (37, 61), others report higher *ERα* expression in comparison to *ERβ* in endometrial cancer tissues (62, 63). The results of this study indicate a differential steroid hormone receptor regulation between RL95-2 and AN3 cells. RL95-2 cells have an increased expression of ERα and ERβ protein during treatments (**Figure 6**). Whereas in AN3 cells, no mRNA data for either were observed and only ERβ protein bands were seen with no statistically significant differences. The results of this study partially agrees with Sun *et al.*, who report significantly increased ERα mRNA and protein expression in AN3 and RL95-2 cells in comparison to other endometrial cancer cell lines (41). They also suggest an increased gene expression of estrogen related receptor alpha (*ERRα*), an orphan nuclear receptor known to mediate the effects of estrogen, in AN3 and RL95-2 cells (41). Therefore implying the need to study the involvement of orphan nuclear receptors in estrogen signaling and action, as well as understanding their association with TETs is imperative. This is also consistent with findings in other endometrial cancer cells that suggest, TET1 increases estrogen sensitivity by upregulating mRNA expression of orphan nuclear receptor - *GPER*, in ishikawa and HEC-1-A cells (64).

PR was not expressed in RL95-2 cells, either at the mRNA level or at the protein level, which is a finding reported in another study as well (65). AR however, was not expressed at the mRNA level but was seen at the protein level, however the differences were not significant (**Figure 6**). Previously, AR protein expression has been reported in endometrial carcinomas with conflicting data on the level of expression. While Sasaki et al., demonstrated hypermethylation mediated AR gene silencing, Ito et al., suggested increased AR expression in endometrial carcinoma tissues (56, 66). Recent data imply that AR positivity is seen in a subset of endometrial carcinomas and is expressed conversely to ERs (55). A similar correlation can be drawn from the findings of this study, wherein increased ERα and ERβ is associated with reduced AR protein expression in RL95-2 cells. Since AR is anti-proliferative, its use as a potential target for curbing uncontrollable cellular hypertrophy is implied however this might not be the case for all tumors and more studies on tumor endocrinology is needed. A better understanding of the steroid hormone regulation and epigenetic axis in female cancers could help in the development of targeted transcriptional endocrine therapies.

In this study, a degree of variation between mRNA and protein expression of TETs and steroid hormone receptors in both, AN3 and RL95-2 cells was observed. The correlation between transcription and translation is complex and depends on several biological and technical factors. It has been suggested that the physical properties of transcription, can alter the translation efficiency at various levels contributing to a discrepancy between mRNA and protein data (67). The other most important and highly variable factor, influencing mRNA-protein correlation is the individual half-lives of proteins (68). For instance, it has been reported that long term estrogen exposure, increases ERα half-life, maintaining protein stability and slowing rate of proteolysis, which could explain the presence of ERα in RL95-2, despite no mRNA expression being observed (69). Subsequently, post-translational and post transcriptional modifications and delayed synthesis between mRNA and protein, could also result in a poor mRNA-protein correlation (70, 71).

In summary, endometrial cancer is complex and involves abnormal steroid hormone signaling. This study evaluates steroid hormone regulation of TETs and steroid hormone receptors *in vitro* and also highlights the importance of evaluating different cancer cell lines independently, to understand the mechanisms of hormone action. It is proposed that differential protein expression of TETs during different hormonal treatments could be involved in the regulation of ERα and ERβ in RL95-2 and AN3 cells. The downregulation AR in AN3 cell line could be explored further as a potential target for hormone therapy. However, for a more comprehensive understanding of the association between TETs and steroid hormone receptors, additional studies including endometrial cancer tissues and primary cells, need to be undertaken. Overall, this study provides a preliminary account, indicating that TETs, steroid hormones and their receptors might be co-regulated to maintain hormone signaling in the endometrium. Future studies involving the assessment of 5-hmC levels and gene promoter sequencing might help in determining the epigenomic regulation of steroid hormone receptors in endometrial cancer cells more definitively.

The protocol used in this study, included a limited 24h of estrogen treatment prior to the addition of a combined estrogen and progesterone. This was done to mimic a snapshot of the molecular events that occur *in utero* during the early proliferative stage. The crucial estrogen priming process, enriches the endometrium with steroid hormone receptors preparing it for a successful progesterone action during the secretory phase. Due to the challenging nature of the tissue and complexity of the experiments, it was not possible to include multiple time points for estrogen priming at this stage but is suggested in the scope for future studies.

## Data Availability Statement

The original contributions presented in the study are included in the article/supplementary material. Further inquiries can be directed to the corresponding author.

## Author Contributions

VM: performed major experiments and primary contributor of manuscript writing and editing manuscript. PG: reviewed manuscript and minor experiments. LJ: reviewed manuscript and minor experiments. AP: conceived the idea, provided perceptive comments on drafts, and approved the content of the manuscript. All authors contributed to the article and approved the submitted version.

## Funding

The research project was funded and supported by Auckland Medical Research Foundation (AMRF, grant number: 1116005). VM was supported by Liggins Institute Departmental Fund and AP was supported by Sidney Taylor Trust, University of Auckland.

## Conflict of Interest

The authors declare that the research was conducted in the absence of any commercial or financial relationships that could be construed as a potential conflict of interest.

## Publisher’s Note

All claims expressed in this article are solely those of the authors and do not necessarily represent those of their affiliated organizations, or those of the publisher, the editors and the reviewers. Any product that may be evaluated in this article, or claim that may be made by its manufacturer, is not guaranteed or endorsed by the publisher.

## Abbreviations

5hmC, 5-hydroxymethylcytosine; 5mC, 5-methylcytosine; AR, Androgen Receptor; AREs, Androgen Responsive Elements; CS-FBS, Charcoal stripped FBS; C treatment, Control; DNMTs, DNA Methyltransferases; E treatment, Estradiol treatment; EP treatment, Estrogen + progesterone treatment; Era, Estrogen receptor alpha; ERβ, Estrogen receptor beta; ER, Estrogen receptors; ERRα, Estrogen related receptor alpha; PR, Progesterone receptor; TDG, thymine DNA glycosylase; TET, Ten-eleven translocation.
